# A Novel, Broad-Acting Peptide Inhibitor of Double-Stranded DNA Virus Gene Expression and Replication

**DOI:** 10.3389/fmicb.2020.601555

**Published:** 2020-11-17

**Authors:** Zsolt Ruzsics, Katja Hoffmann, André Riedl, Adalbert Krawczyk, Marek Widera, Helene Sertznig, Leonie Schipper, Valeria Kapper-Falcone, Monika Debreczeny, Wolfgang Ernst, Reingard Grabherr, Hartmut Hengel, Hanna Harant

**Affiliations:** ^1^Institute of Virology, Medical Center-University of Freiburg, Freiburg, Germany; ^2^Faculty of Medicine, University of Freiburg, Freiburg, Germany; ^3^German Consulting Laboratory for HSV and VZV, Medical Center-University of Freiburg, Freiburg, Germany; ^4^Department of Infectious Diseases, West German Centre of Infectious Diseases, Universitätsmedizin Essen, University Duisburg-Essen, Essen, Germany; ^5^Institute for Virology, University Hospital Essen, University of Duisburg-Essen, Essen, Germany; ^6^Institute of Medical Virology, University Hospital Frankfurt, Goethe University Frankfurt, Frankfurt, Germany; ^7^VIBT Imaging Center, University of Natural Resources and Life Sciences, Vienna, Austria; ^8^Department of Biotechnology, University of Natural Resources and Life Sciences, Vienna, Austria; ^9^Pivaris BioScience GmbH, Vienna, Austria

**Keywords:** antiviral peptide, herpes simplex virus, cytomegalovirus, adenovirus, polyomavirus

## Abstract

Viral infections are a global disease burden with only a limited number of antiviral agents available. Due to newly emerging viral pathogens and increasing occurrence of drug resistance, there is a continuous need for additional therapeutic options, preferably with extended target range. In the present study, we describe a novel antiviral peptide with broad activity against several double-stranded DNA viruses. The 22-mer peptide TAT-I24 potently neutralized viruses such as herpes simplex viruses, adenovirus type 5, cytomegalovirus, vaccinia virus, and simian virus 40 in cell culture models, while being less active against RNA viruses. The peptide TAT-I24 therefore represents a novel and promising drug candidate for use against double-stranded DNA viruses.

## Introduction

Effective therapeutic intervention by antiviral agents is still limited to specific viral infections, such as those caused by influenza A virus, herpes viruses, including herpes simplex virus (HSV) and cytomegalovirus (CMV), as well as hepatitis B and C. Antiviral drugs for treatment of herpes virus infections include nucleoside analogues, such as aciclovir (Elion et al., 1977; Schaeffer et al., 1978) and derivatives (De Clercq and Field, 2006), pyrophosphate analogues, such as foscavir (Helgstrand et al., 1978), or primase inhibitors such as pritelivir (Kleymann et al., 2002), BILS 179 BS (Crute et al., 2002), and amenavir (Chono et al., 2013). Letermovir, a viral terminase complex inhibitor, is used for the prevention of CMV infections in hematopoietic stem cell transplant patients (Marty et al., 2017). Strenuous efforts have been invested in the treatment of infections by human immunodeficiency virus-1 (HIV-1) with various efficient antiviral agents available (Littler and Oberg, 2005; De Clercq and Li, 2016). These include reverse transcriptase, integrase, and protease inhibitors (Mitsuya et al., 1985; Roberts et al., 1990; Hazuda, 2000; Sato et al., 2006; Raffi et al., 2013). One example of a peptide drug is the viral fusion inhibitor enfuvirtide (Fuzeon®), an approved drug for treatment of HIV (Matthews et al., 2004).

However, the majority of viruses cannot be targeted by an antiviral agent, and for many of those no suitable vaccine is available. There are several reasons for limitations in the development of antiviral agents. Most drugs, which act specifically against one virus cannot be used for treatment of other viral diseases since the relevant drug targets are virus-specific. Other limitations include long-term toxicity of certain drugs as well as development of resistance against a drug. Broad-acting antiviral agents, which target several viruses could therefore be of high medical and also economic interest, but would require extensive development (Dropulic and Cohen, 2010; Zhu et al., 2015). Such a broad-acting drug could target either a host molecule indispensable for viral infections or viral target molecules sufficiently conserved between several viruses. One example of a potential broad-acting inhibitor is CMX-100 (brincidofovir), which can inhibit replication of several DNA viruses such as herpes viruses, poxviruses, adenoviruses, and polyomavirus (Beadle et al., 2002).

In this study, we describe the discovery of a novel, 22-mer peptide, which has the ability to inhibit gene expression and replication *in vitro* of double-stranded DNA viruses of multiple and diverse taxa, including baculovirus infecting mammalian cells, adenovirus type 5, herpes simplex viruses, cytomegalovirus, SV40 polyomavirus, and vaccinia virus.

## Materials and Methods

### Plasmids

The firefly luciferase coding region was cloned into pcDNA3.1 (ThermoFisher). The truncated interleukin-8 (CXCL8) promoter upstream of the firefly luciferase coding region cloned into pGL2-basic vector has been described previously (Harant et al., 1996). Plasmids were purified from *Escherichia coli* cultures using Wizard® Plus Midiprep DNA Purification System (Promega).

### Peptides

Peptides were synthesized at JPT Peptide Technologies (Berlin, Germany) or Bachem AG (Switzerland). The peptides CLAFYACFC (I24), GRKKRRQRRRPPQ (TAT 48–60), and GRKKRRQRRRPPQCLAFYACFC (TAT-I24) were purified with HPLC to >90% purity. Where indicated, TAT peptide from Sigma was used (TAT 47–57; YGRKKRRQRRR). The peptides SV40 NSL (PKKKRKVEDPY), SV40 NSL-I24 (PKKKRKVEDPYCLAFYACFC), and TAT-C (YGRKKRRQRRRC) were synthesized at JPT Peptide Technologies. The peptide FAM-TAT consists of the TAT peptide (48–60) labeled with 6-carboxyfluorescein at the N-terminal end and was synthesized at JPT Peptide Technologies. The peptide FAM-TAT-I24 consists of a fusion of TAT (47–57) and I24 labeled with 6-carboxyfluorescein at the N-terminal end and was synthesized at Bachem AG. Peptides were dissolved in DMSO (Sigma-Aldrich, St. Louis, MO, United States) as 10 mM stock and stored at −20°C.

### Cell Culture

Vero, MRC-5, and CV-1 cells were cultured in Dulbecco’s Modified Eagle Medium (DMEM; ThermoFisher, Waltham, MA, United States) and Jurkat cells were cultured in RPMI 1640 medium containing 10% fetal calf serum, 100 U/ml penicillin, and 0.1 mg/ml streptomycin (ThermoFisher). HEK293 and NIH/3T3 cells were adapted to growth in CO_2_-independent medium supplemented with 10% fetal calf serum, 2 mM glutamine, and 1% antibiotic-antimycotic (ThermoFisher) and cultivated in a humidified atmosphere at 37°C. For analysis of cell viability, HEK293 cells were seeded at a density of 1 × 10^4^ cells/well of a 96-well plate and incubated with peptides for 96 h. Viability was analyzed using CellTiter-Glo® 2.0 Assay according to the manufacturer’s protocol (Promega). For determination of cytotoxicity, NIH/3T3 cells were seeded at a density of 2 × 10^4^ cells/well of a white 96-well plate and incubated with peptide dilutions for 72 h. Cytotoxicity was analyzed using MultiTox-Glo Multiplex Cytotoxicity Assay according to the manufacturer’s protocol (Promega).

### DNA Transfections

HEK293 cells were seeded at a density of 1.2 × 10^5^ cells/well of a 48-well plate and transfected on the next day with plasmid DNA and Superfect transfection reagent (Qiagen) in the presence of peptides or DMSO vehicle control. Briefly, for one well of a 48-well plate, 100 ng DNA and 1 μl Superfect reagent were mixed by pipetting followed by addition of 12.5 μl of serum‐ and antibiotic-free medium. After brief vortexing and further incubation at room temperature for 15 min, 112.5 μl medium containing 10% fetal calf serum and antibiotics were added. For transfection in the presence of peptides, a transfection mixture of DNA, Superfect reagent, and serum-and antibiotic-free medium were prepared for the required number of wells. From this mixture, 25 μl aliquots were made and 0.5 μl peptides (10 mM each) added to each aliquot, vortexed briefly, and incubated for 15 min at room temperature. Then, 225 μl of medium containing 10% fetal calf serum was added and 125 μl applied per well of duplicate wells. For other types of multi-well plates, volumes were adapted according to the growth area of the wells. After 24 h, cells were lysed using 20 μl/well of Luciferase Cell Culture Lysis Reagent (Promega) and subjected to luciferase determination using 10 μl lysate and 50 μl of Luciferase Assay System (Promega) and the GloMax Multi instrument (Promega).

Levels of CXCL8 in the supernatants were measured by stimulating cells with tumor necrosis factor-α (TNF-α; ThermoFisher) 6 h after transfection and harvesting supernatants 18 h later. CXCL8 levels were then determined using a human interleukin-8 ELISA according to the manufacturers´ instructions (Human IL-8 ELISA Set, Diaclone). For RNA analysis, HEK293 cells were seeded at a density of 2.4 × 10^5^ cells/well of a 24-well plate and transfected with pcDNA3.1-luciferase plasmid. Six hours after transfection, cells were stimulated with TNF-α for further 18 h before isolation of total RNA as described below.

### RNA Isolation and Real-Time PCR

Oligonucleotides were synthesized at Microsynth AG (Balgach, CH). RNA was isolated using RNeasy Mini kit (Qiagen). Cells were lysed with RLT buffer and total RNA eluted with 50 μl of nuclease-free water. A total of 17.5 μl of the eluates were subjected to DNAse I digestion to remove plasmid or viral DNA using RNase-Free DNase Set (Qiagen) in a 20 μl reaction for 30 min at 37°C followed by heat inactivation at 80°C for 10 min. Synthesis of cDNA was then performed with 9 μl of DNAse-digested RNA using High-Capacity cDNA Reverse Transcription Kit (Applied Biosystems/ThermoFisher) in a 18 μl reaction volume according to the manufacturer’s instructions. The cDNA was then diluted to 1:2 with nuclease-free water and real-time PCR was performed with 2 μl cDNA per 20 μl PCR reaction. Real-time PCR was used for the detection of luciferase and utilized the primers luciferase for: 5'-TGGAGAGCAACTGCATAAGG-3' (600 nM), luciferase rev: 5'-CGTTTCATAGCTTCTGCCAA-3' (600 nM), the luciferase probe: 5'-FAM-ACGCCCTGGTTCCTGGAACAA-TAMRA-3' (200 nM), and TaqMan™ Universal PCR Master Mix (Applied Biosystems/ThermoFisher). For the detection of human GAPDH and human IL-8 (CXCL8), premixed assays from ThermoFisher were used (GAPDH: #4310884E; CXCL8: #Hs00174103). The adenovirus hexon gene was quantified by real-time PCR using ViroReal® Adenovirus (RTGM33RV, Ingenetix GmbH, Austria) and Taqman™ Universal PCR Mastermix, and a plasmid standard containing the amplicon region of adenovirus 5 cloned into pCR2.1. Human CMV UL44 transcripts were detected using ViroReal® CMV (RTGM10V, Ingenetix GmbH, Austria) and Taqman™ Fast Universal PCR Mastermix (Applied Biosystems/ThermoFisher). Herpes simplex virus was detected using the gene-specific primer pairs: HSV-1 UL30 for: 5'-CATCACCGACCCGGAGAGGGAC-3' and HSV-1 UL30 rev: 5'-GGGCCAGGCGCTTGTTGGTGTA-3' according to Kessler et al. (2000) and normalized to 18S rRNA using primers 18S for: 5'-GTAACCCGTTGAACCCCATT-3', and 18S rev: 5'-CCATCCAATCGGTAGTAGCG-3' according to Korom et al. (2008). Large T antigen from SV40-infected cells was assessed using gene-specific primers LT for: 5'-GTTTCAGGTTCAGGGGGAGG-3' and LT rev: 5'-TCAGGGCATGAAACAGGCAT-3' and normalized to 18S rRNA. For HSV and SV40, Luna® Universal qPCR Mastermix (New England BioLabs) and 250 nM of gene-specific forward and reverse primer were used. All real-time PCR runs were performed using an Applied Biosystems® 7500 Fast Real-Time PCR System (ThermoFisher).

### Viruses

#### Baculovirus

A baculovirus expressing firefly luciferase was generated by cloning the luciferase coding region under control of the CMV promoter into pACEBac1 and the MultiBac system. Baculovirus was then propagated in Sf9 insect cells and supernatants of infected cells were collected after 4–5 days, containing 5 × 10^7^–1 × 10^8^ plaque forming units/ml. Supernatants were directly used to infect HEK293 cells which were seeded at 1.2 × 10^5^ cells in each well of a 48-well plate. On the next day, peptides were added at a 1:500 dilution (starting from 10 mM stock solution) in culture medium to the cells directly, followed by infection of the cells with baculovirus at a multiplicity of infection (MOI) of 5. After 24 h, cells were lysed with 20 μl/well of Luciferase Cell Culture Lysis Reagent (Promega) and luciferase recorded using 10 μl lysate and 50 μl of Luciferase Assay System (Promega) and a GloMax Multi instrument (Promega). Luciferase was normalized to total protein content of the lysates determined using Pierce BCA Protein Assay Kit (ThermoFisher). For analysis of the time of peptide addition, 2.5 × 10^4^ cells were seeded into each well of a white 96-well plate and infected on the next day with Baculovirus-Luc at a MOI of 5 and treated with increasing concentrations of TAT-I24 either simultaneously or after 3, 6, and 24 h. Cells were lysed 48 h post-infection in 10 μl/well of Luciferase Cell Culture Lysis Reagent and luciferase recorded using 50 μl/well Luciferase Assay System. For RNA analysis, 1 × 10^5^ cells were seeded per well of a 12-well plate, treated with peptides and infected with Baculovirus-Luc (MOI = 2.5). Six hours after infection, cells were either left unstimulated or stimulated with TNF-α (10 ng/ml) for 18 h before isolation of total RNA using RNeasy Mini Kit (Qiagen) as described above.

#### Adenovirus

Adenoviral particles expressing luciferase/GFP (#AVP004) were purchased from AMSBIO (United Kingdom) containing 1 × 10^8^ infectious units/ml. Luciferase activity from infected HEK293 cells was recorded using Steady Glo® Luciferase Assay System (Promega). For RNA analysis, HEK293 cells were seeded at a density of 1 × 10^5^ cells/well of a 12-well plate, treated with peptides and infected with adenovirus particles (MOI = 1). After 6 h, cells were either left untreated or stimulated with TNF-α (10 ng/ml) for 18 h before isolation of total RNA using RNeasy Mini Kit (Qiagen) as described above.

Adenovirus replication was analyzed as follows: HEK293 cells were seeded at a density of 4 × 10^4^ cells/well of a 12-well plate. On the next day, cells were treated with peptides at the indicated time points and infected with adenovirus (MOI of 0.5) in a volume of 800 μl medium. After 6 days, viral DNA was extracted from 70 μl of the supernatants using QIAamp Viral RNA Mini kit (Qiagen), which also allows purification of viral DNA (Lackner et al., 2014). Viral DNA was eluted with 60 μl elution buffer. Adenovirus DNA was then quantified by real-time PCR from 5 μl eluates per PCR reaction and a quantitative plasmid standard as described above.

#### Murine Cytomegalovirus

NIH/3T3 cells were seeded at a density of 2 × 10^4^ cells/well of a 96-well plate and allowed to attach overnight. On the next day, medium was removed and replaced by medium containing peptide dilutions. Then, murine CMV (MCMV) expressing firefly luciferase (Trilling et al., 2011) was added to the cells at a MOI of 0.5 with aid of centrifugal enhancement at 800 × *g* for a total of 30 min. After 72 h, cells were lysed using 20 μl/well of Luciferase Cell Culture Lysis Reagent (Promega) and luciferase recorded from 10 μl using 50 μl Luciferase Assay System (Promega).

Virus adsorption was performed by addition of MCMV expressing luciferase at a MOI of 0.5 with centrifugal enhancement at +4°C and further incubation at +4°C for 60 min before removal of unabsorbed virus and three times washing of cells with cold medium. Cells were then transferred to the incubator and kept at 37°C. Peptide was added at different time points before or after virus adsorption. After 72 h incubation at 37°C, luciferase levels were determined from cell lysates as described above.

In another setting, NIH/3T3 cells were preincubated with TAT-I24 at concentrations of 1 and 10 μM for 1, 3, and 6 h followed by removal of peptide and 3x washing with medium. Cells were then infected with MCMV expressing luciferase with the aid of centrifugal enhancement. After 72 h, luciferase levels were determined from cell lysates as described above.

#### Herpes Simplex Virus, Human Cytomegalovirus, and Adenovirus-5 Replicon Assays

The virus strains used were HSV strains HSV-1 F (Ejercito et al., 1968) and the aciclovir (ACV)-resistant HSV-1 clinical isolate 703 and the aciclovir-sensitive clinical isolate HSV-1601 (both control strains for resistance testing by the German Consulting Laboratory for HSV and VZV, University Medical Center Freiburg). Replicon assays for HSV, adenovirus 5, and human cytomegalovirus (HCMV) TB40 were performed with the cell lines Vero, A549, and MRC-5, respectively (Koszinowski et al., 2016). Cells were seeded in 96-well plates and infected with AAV-GLuc-3B (Sirion Biotech, Germany) with 1,500 genome copies/cell for Vero and A549 cells and 10,000 genome copies for MRC-5 cells. After 24 h, cells were preincubated with peptide dilutions for 1 h at 37°C before infection with HSV-1 isolates at a MOI of 0.035, and adenovirus 4, adenovirus 5, or adenovirus 19a/64 (provided by Albert Heim, Institute for Virology, German National Reference Laboratory for Adenoviruses, Hannover Medical School, Germany) at a MOI 1.0 or HCMV TB40 at a MOI 0.2. Gaussia luciferase released into the medium was determined after 24 h for HSV-1 and adenovirus or after 3 days for HCMV. For subsequent RNA analysis, cells were directly lysed using 100 μl of RLT buffer and RNA extracted using RNeasy Mini Kit (Qiagen) and real-time PCR analysis as described above.

#### Herpes Simplex Virus Neutralization Assay

Herpes simplex virus (HSV) strains HSV-1 F and HSV-2 G were propagated in Vero cells as previously described (Krawczyk et al., 2013). Viral titres were determined by a standard endpoint dilution assay and calculated as 50% tissue culture infectious dose (TCID_50_)/ml as previously described (Reed and Muench, 1938). To investigate whether TAT-I24 can completely neutralize HSV-1 F or HSV-2 G, a standard endpoint dilution assay was performed as previously described (Krawczyk et al., 2010; Zinser et al., 2018). Serial dilutions of TAT-I24 (10–0.08 μM) were pre-incubated with 100 TCID_50_ of HSV-1 F or HSV-2 G at 37°C for 1 h. Subsequently, virus/TAT-I24 mixtures were applied to Vero cells seeded in 96-well plates (10 wells per dilution). The cytopathic effect (CPE) was scored after 48 h of incubation at 37°C. The concentration of TAT-I24 required for reducing the virus-induced CPE by 100% was defined as the complete neutralization titer.

#### Vaccinia Virus

CV-1 cells were seeded at a density of 7 × 10^4^ cells/well of 48-well plates and allowed to attach 4 h before infection with vaccinia virus wild-type (wt; strain Western Reserve) at a MOI of 0.2. Cells were preincubated with peptide dilutions for 1 h at 37°C before infection with vaccinia virus (VACV wt). Twenty-four hours post-infection, plates were subjected to one freeze (−80°C) and thaw cycle. Supernatants of lysed cells were then titrated by limiting dilution on CV-1 cells seeded 4 h before infection into 96-plates at a density of 4 × 10^4^ cells/well. Vaccinia virus plaques were counted and TCID_50_ calculated by the Kärber statistical method (Kärber, 1931).

#### Simian Virus 40

Vero cells were seeded into 96-well plates 1 day before infection with simian virus 40 (SV40) strain VR-239 (ATCC) at a MOI of 2. After 3 days, total RNA was isolated, digested with DNAse I, and cDNA then synthesized. Transcripts of large T antigen (LT) were quantified by real-time PCR as described above.

#### Human Immunodeficiency Virus-1

Human immunodeficiency virus-1 (HIV-1) virus stocks were generated as described previously (Widera et al., 2014a). Jurkat T-cells were seeded at a density of 1 × 10^5^ cells/well of 96-well plates and incubated overnight. The HIV-1 lab strain NL4-3 (Adachi et al., 1986) or medium only were pre-incubated with peptides at different concentrations for 1 h at 37°C before infection of cells (0.05 MOI). After 6 days, supernatants of infected Jurkat cells were used to infect TZM-bl reporter cells (Wei et al., 2002), which were assayed for luciferase or stained with X-Gal 48 h post-infection as described previously (Widera et al., 2014b).

### Microscopy

Localization studies employed NIH/3T3 cells, seeded at a density of 4 × 10^4^ cells/well into poly-l-lysine treated eight-well chambers (ibidi, Germany) and allowed to attach for 24 h. On the next day, FAM-TAT peptide was added directly to the culture medium. The peptide FAM-TAT-I24 was first diluted in 1/10 of the final volume in PBS and then added to culture medium followed by centrifugation in a microcentrifuge at 13,000 rpm for 5 min to remove any precipitate. After 1 h, cells were fixed with 5% formaldehyde for 10 min followed by staining of the nuclei with 4',6-diamidin-2-phenylindol (DAPI) for 15 min.

For analysis of MCMV replication, NIH/3T3 cells were seeded at a density of 2 × 10^4^ cells/well of ibiTreat eight-well chambers (ibidi, Germany). On the next day, cells were treated with peptides and infected with murine CMV S-mCherry-SCP (Bosse et al., 2012) at a MOI 0.2 for 72 h before fixation with 5% formaldehyde and staining of nuclei with DAPI. Microscopic examination was performed using a Live Cell Video Microscope (Leica Microsystems).

For analysis of MCMV entry, NIH/3T3 cells were seeded at a density of 4 × 10^4^ cells/well into ibidi-Treat well chambers (ibidi, Germany) and treated on the next day with 10 μM TAT-I24 or left untreated and infected with S-mCherry-SCP at a MOI of 15. Virus was adsorbed at 4°C with the aid of centrifugal enhancement. After 1.5 h at +4°C, unbound virus was removed from the cells by washing with ice-cold medium followed by a temperature shift to +37°C. After 15 and 60 min at +37°C, cells were washed three times with PBS and then fixed with 5% formaldehyde followed by staining with DAPI. Microscopic examination was performed using a Live Cell Video Microscope (Leica Microsystems).

### Data Analysis

EC_50_ values were calculated with non-linear curve fitting tools using GraphPad Prism 8 (GraphPad Software, San Diego, United States).

## Results

### Selective Inhibition of Reporter Gene Expression From Transfected Plasmid DNA by a Novel Peptide

**Figure 1:**
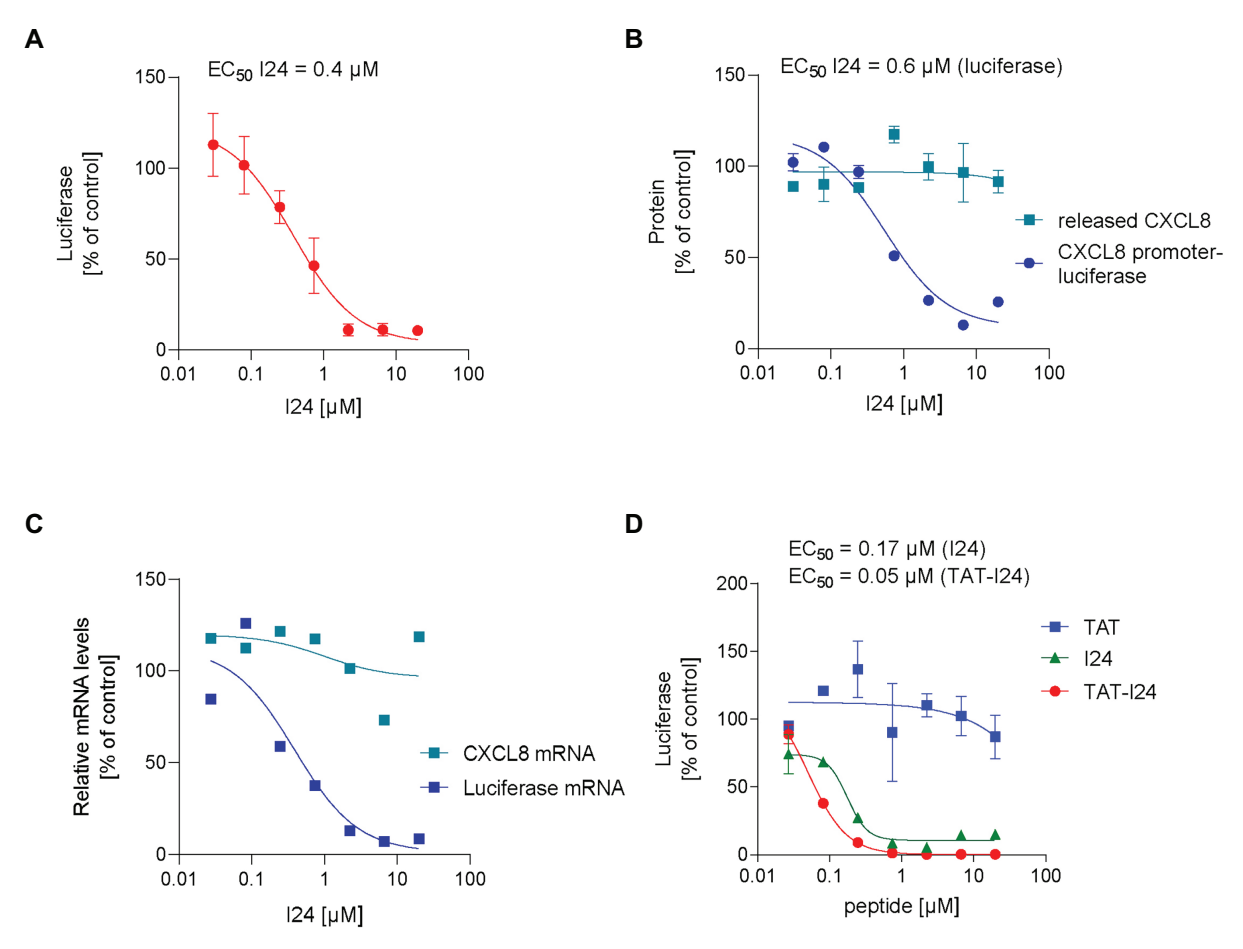
Selective inhibition of plasmid-encoded gene expression by I24. **(A)** HEK293 cells were transfected with an expression plasmid encoding firefly luciferase under control of the CMV promoter in the presence of increasing concentrations of peptide I24. Results shown are luciferase levels (mean ± SD from triplicate wells). **(B)** HEK293 cells were transfected with a truncated IL-8 (CXCL8) promoter-luciferase expression construct in the presence of increasing concentrations of I24 and treated with TNF-α 6 h after transfection for further 18 h. Luciferase was determined from cell lysates and supernatants analyzed for CXCL8 by ELISA. Results shown are mean ± SD from duplicate wells expressed as % of vehicle-treated control. **(C)** Total RNA was isolated from cells transfected with the luciferase expression plasmid and stimulated with TNF-α 6 h after transfection for further 18 h before isolation of total RNA. Results shown are luciferase and CXCL8 mRNA levels normalized to GAPDH mRNA expressed as % of vehicle-treated control. **(D)** HEK293 cells were transfected with the luciferase expression plasmid and increasing concentrations of I24, TAT (47–57), or TAT-I24. Luciferase levels (mean ± SD from duplicate wells) expressed as % of vehicle-treated cells are shown.

The peptide I24 is a linear, 9-mer peptide with the sequence CLAFYACFC, which was shown to selectively inhibit gene expression from a “foreign” DNA (Harant, 2017). Transfection of HEK293 cells with a plasmid containing the firefly luciferase reporter gene under control of the CMV promoter in the presence of increasing concentrations of I24 caused a dose-dependent inhibition of luciferase production. The extent of inhibition was about 90% at the highest concentration tested (20 μM) with a calculated EC_50_ of 0.4 μM compared to luciferase levels in vehicle-treated cells (Figure 1A). Inhibition of reporter gene expression was seen only when the peptide was added together with the DNA to the transfection complex, suggesting a possible direct interaction of the peptide with the plasmid DNA. The transfection procedure used in these experiments is based on endocytosis by activated dendrimers, which enable efficient transfection (Tang et al., 1996). An inhibitory effect – although less pronounced – was also observed using lipofection. It is possible that the lipofection impacts the activity of the peptide due to its hydrophobicity (Supplementary Figure S1).

Inhibition of luciferase reporter gene expression was also seen when the luciferase coding region was expressed under control of a cytokine-inducible promoter, such as the truncated promoter of CXCL8 (interleukin-8) as described previously (Harant et al., 1996). Transfection with this plasmid in the presence of increasing concentrations of peptide I24 and further stimulation with TNF-α dose-dependently decreased luciferase levels with an EC_50_ of 0.6 μM (Figure 1B). In parallel, levels of CXCL8, expression of which is highly induced by TNF-α, were analyzed from supernatants from the same wells 24 h after transfection. Independent of the peptide concentrations used, levels of CXCL-8 in the supernatants were unaffected by the peptide, indicating that expression of endogenous, cellular genes, is not inhibited by the peptide to the same extent (Figure 1B).

Inhibition of reporter gene expression was observed at the level of mRNA expression. HEK293 cells were transfected with the luciferase expression plasmid and stimulated with TNF-α (10 ng/ml) 6 h after transfection, and the total RNA was isolated 24 h after transfection. RNA was then treated with DNase I for 30 min to remove residual plasmid DNA before synthesis of a cDNA using random hexamers. For relative quantification, luciferase transcripts were normalized to the GAPDH housekeeping gene. Levels of luciferase mRNA were downregulated by up to 90% in HEK293 cells transfected with plasmid DNA in the presence of peptide I24. However, levels of endogenous, TNF-α-induced CXCL8 transcripts were not affected by the peptide (Figure 1C).

### Inhibition of Baculovirus Gene Expression in Mammalian Cells by TAT-I24

To evaluate any potential effect of this peptide on a DNA delivered by viral transduction, a baculovirus was employed. Baculovirus (*Autographa californica* multiple nucleopolyhedrosis virus) is an enveloped insect virus with a double-stranded DNA genome and can also transduce a wide range of mammalian cell lines. Baculovirus therefore represents a useful gene delivery system for generation of recombinant proteins. Furthermore, baculoviruses cannot replicate in mammalian cells making them attractive as protein expression system (Mansouri and Berger, 2018). To test an effect of the peptide on viral transduction, a baculovirus was generated, which expresses luciferase under control of a CMV promoter (Baculovirus-Luc). To overcome a potential delivery problem, a cell penetrating version of the peptide was generated by fusion of the HIV-TAT peptide (48–60) at the N-terminal end of the peptide I24, now named TAT-I24. When the peptide TAT-I24 was applied to a transfection reaction with plasmid DNA as described above, reporter gene expression was inhibited more potently compared to I24, while the TAT peptide alone was ineffective (Figure 1D). These data demonstrate that fusion of I24 with the TAT peptide enhances cell penetration and/or potency of the peptide.

**Figure 2:**
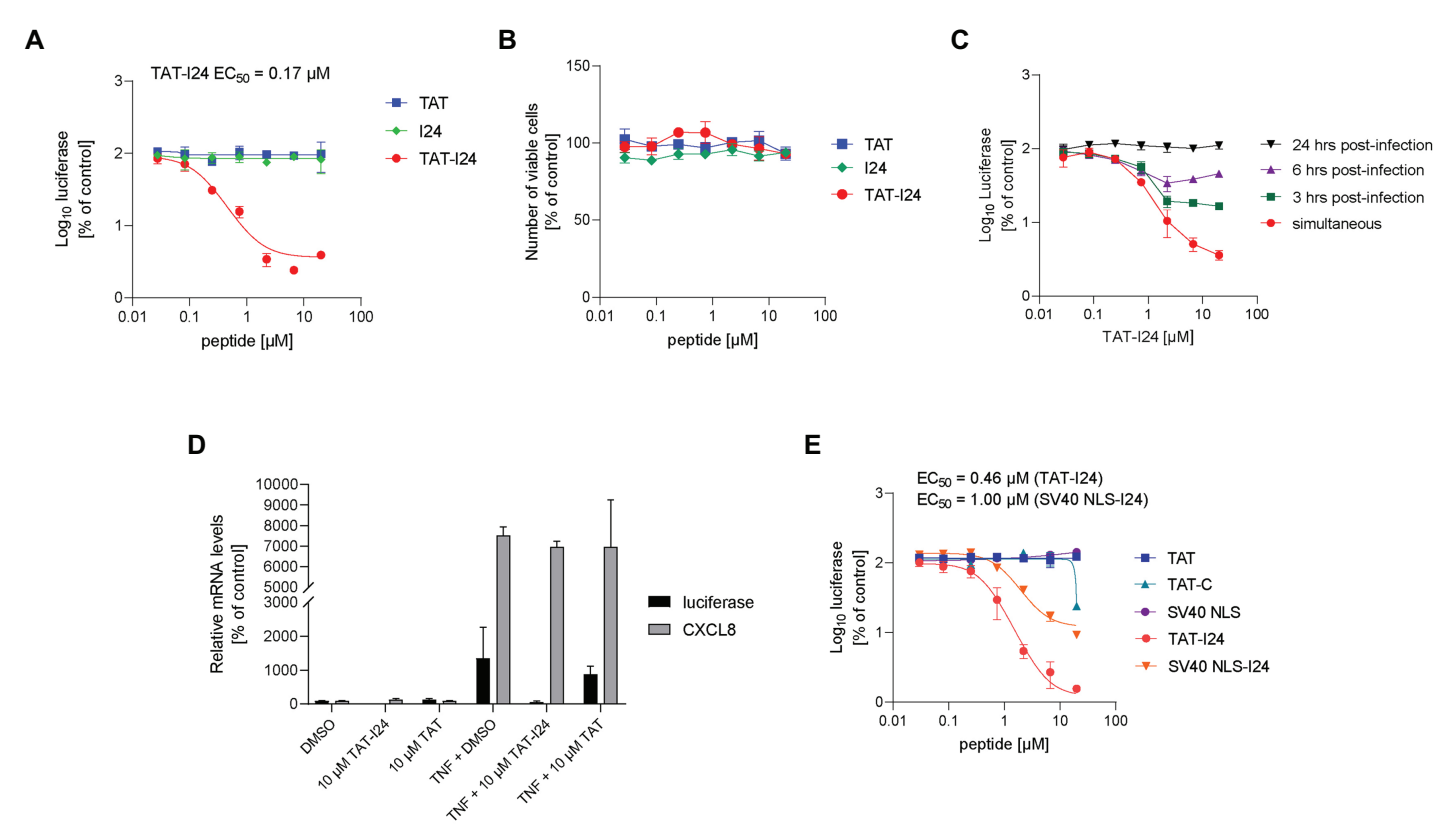
Inhibition of reporter gene expression from baculovirus by TAT-I24. **(A)** Luciferase levels (mean ± SD from duplicate wells) relative to vehicle-treated controls from HEK293 cells treated with I24, TAT (47–57), or TAT-I24 and transduced with Baculovirus-Luc for 24 h. **(B)** Cell viability (luciferase mean ± SD from triplicate wells) of HEK293 cells treated with TAT (48–60), TAT-I24, or I24 for 96 h. **(C)** HEK293 cells were infected with Baculovirus-Luc and treated with TAT-I24 dilutions either simultaneously or 3, 6, or 24 h after infection. Cells were lysed 48 h after infection. Luciferase levels (mean ± SD from triplicate wells) relative to vehicle-treated cells are shown. **(D)** luciferase and CXCL8 mRNA levels relative to GAPDH mRNA from duplicate extractions of HEK293 cells treated with 10 μM TAT (48–60), TAT-I24, or DMSO and infected with Baculovirus-luc and stimulated after 6 h with TNF-α (10 ng/ml) for 18 h. Data are expressed as % of vehicle-treated control. **(E)** Luciferase levels (mean ± SD from triplicate wells) relative to vehicle-treated controls from HEK293 cells treated with TAT (48–60), TAT-C, SV40 NLS, TAT-I24, or SV40 NLS-I24 and transduced with Baculovirus-Luc.

To examine the effect on reporter gene expression in baculovirus-transduced cells, peptides were directly applied to HEK293 cells subsequently infected with Baculovirus-Luc. However, in contrast to transfection with plasmid DNA, luciferase levels were not affected by I24, indicating that this peptide is not able to inhibit reporter gene expression from a virus-delivered DNA. It was therefore speculated that under these conditions either delivery of the peptide to the cells without transfection reagent is insufficient for inhibition of reporter gene expression or the peptide is not contacting the baculovirus DNA within the cells. Indeed, TAT-I24, the fusion of I24 to the TAT peptide, dose-dependently inhibited reporter gene expression by >95% with an EC_50_ of 0.17 μM when added together with baculovirus. However, the TAT peptide alone did not affect luciferase expression (Figure 2A). At concentrations of TAT-I24 >10 μM, some morphological changes in HEK293 cells were observed. To exclude potential cytotoxicity, all peptides were tested in a cell viability assay in HEK293 cells. The peptides I24, TAT, and TAT-I24 had no effects on HEK293 cell viability after incubation for a period of 96 h (Figure 2B).

To determine the relevance of the time of peptide addition, HEK293 cells were infected with baculovirus and TAT-I24 was added either simultaneously or at different time-points after infection. As shown in Figure 2C, TAT-I24 inhibited luciferase gene expression when added simultaneously while the extent of inhibition was reduced when added 3 or 6 h after infection. No inhibition was observed when the peptide was added 24 h after virus infection, indicating that the peptide is only active during early stages of virus infection (Figure 2C).

The inhibitory effect was also reflected at the level of mRNA expression, as luciferase mRNA levels were inhibited by TAT-I24 relative to the housekeeping gene GAPDH. However, endogenous TNF-α-induced CXCL8 (interleukin-8) mRNA expression was unaffected, indicating that inducible host gene expression is not inhibited to the same extent (Figure 2D).

The peptide I24 was also fused to other cell penetrating peptides. Fusion of I24 to the SV40 NLS peptide inhibited reporter gene expression with an EC_50_ of 1 μM, while the SV40 NLS peptide alone had no effect. However, the overall extent of inhibition by SV40 NLS-I24 was lower compared to TAT-I24 (Figure 2E).

The TAT peptide has been reported to have an antiviral effect against herpes simplex virus (Bultmann and Brandt, 2002; Akkarawongsa et al., 2008). Moreover, addition of a C-terminal cysteine residue to the TAT peptide can enhance its antiviral activity (Bultmann et al., 2007). To distinguish between the effects of TAT containing a C-terminal cysteine residue (TAT-C) and TAT-I24, both peptides were compared for their inhibition of luciferase expression using the baculovirus system. While TAT-I24 potently inhibited luciferase expression, TAT-C caused only partial inhibition at the highest tested concentration of 20 μM (Figure 2E).

### TAT-I24 Inhibits Reporter Gene Expression and Replication of Adenovirus Type 5

To establish the inhibitory potential in the context of a permissive viral target, an infection with adenovirus type 5, a non-enveloped double-stranded DNA virus, expressing firefly luciferase was employed. This system is suitable for gene transduction, as the virus is replication-incompetent due to deletions of the E1 and E3 genomic regions. However, the virus is able to replicate in HEK293 cells, as these cells provide the E1 region due to transformation with adenovirus 5 DNA (Graham et al., 1977).

**Figure 3:**
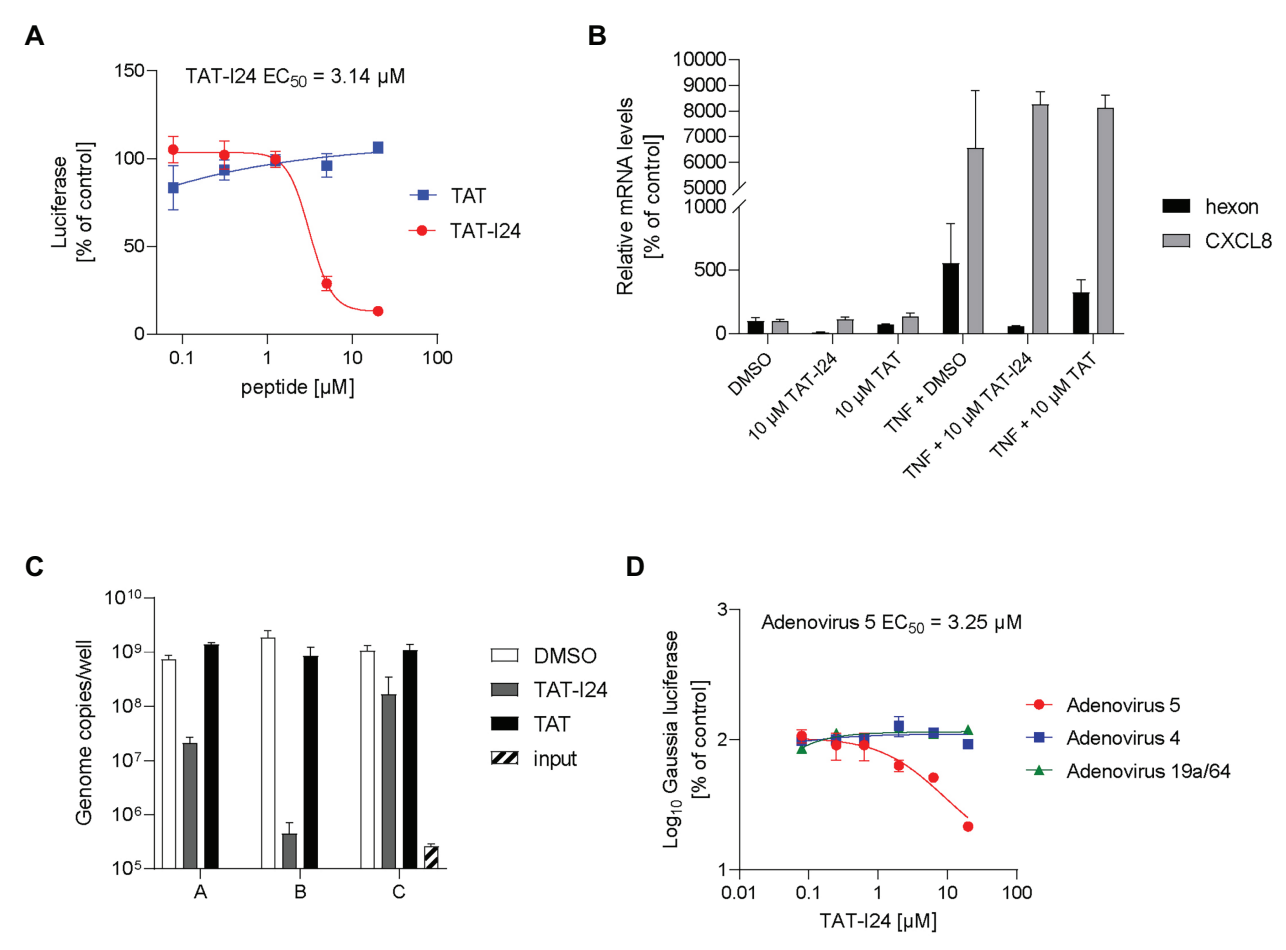
Selective inhibition of gene expression and replication of adenovirus type 5 by TAT-I24. **(A)** luciferase levels (mean ± SD from duplicate wells) of HEK293 cells treated with TAT (48–60) or TAT-I24 and infected with adenovirus-GFP-Luc vector for 24 h. **(B)** Relative hexon and CXCL8 mRNA levels from duplicate extractions of HEK293 cells treated with 10 μM TAT (48–60), TAT-I24, or DMSO and infected with adenovirus-GFP-Luc and with TNF-α (10 ng/ml) stimulation after 6 h for further 18 h. Data are expressed as % of vehicle-treated control. **(C)** Adenovirus genome copies from supernatants from duplicate treatments of infected HEK293 cells with a single dose of 10 μM TAT-I24 for 6 days (group A), with a single dose of 10 μM TAT-I24, and two additional doses of 5 μM TAT-I24 on days 2 and 4 (group B) or treated with 10 μM TAT-I24 24 h post-infection and two additional doses of 5 μM TAT-I24 on days 2 and 4 (group C). **(D)** Gaussia luciferase levels in the supernatants (mean ± SD from triplicate wells) expressed as % of vehicle-treated control from A549 cells transduced with recombinant reporter construct AAV-GLuc-3B and treated on the next day with increasing concentrations of TAT-I24 and infected with adenovirus 4, 5, or 19a/64.

Inhibition of reporter gene expression occurred at higher peptide concentrations compared to baculovirus (EC_50_ = 3–6 μM), while the TAT peptide alone was again ineffective (Figure 3A). As with baculovirus, only TAT-I24 was able to inhibit reporter gene expression, while I24 without the TAT fusion partner was ineffective (Supplementary Figure S2). Analogous to baculovirus, mRNA levels of the virus-encoded hexon gene were downregulated relative to the housekeeping gene GAPDH in the presence of TAT-I24, while expression of TNF-α-induced CXCL8 mRNA levels remained unaffected (Figure 3B).

The effect of peptides on adenovirus replication in HEK293 cells was assessed 6 days after infection. Viral particles released into the supernatant were quantified by real-time PCR analysis of the hexon gene from extracted DNA. In the presence of 10 μM TAT-I24, the total number of genome copies released into the medium was reduced by >97% (Figure 3C, group A). When the peptide was added once at 10 μM before infection combined with subsequent doses of 5 μM each after 2 and 4 days, viral genome counts in the supernatants were reduced by >99% (Figure 3C, group B). However, when 10 μM TAT-I24 was added 24 h after infection followed by additions of 5 μM peptide on days 2 and 4, the extent of inhibition was reduced (84% compared to vehicle control). This observation indicates that the peptide was less effective when added after initial infection but could still inhibit adenovirus spread (Figure 3C, group C).

Finally, the effect of TAT-I24 was confirmed with a wild-type adenovirus 5 strain using a replicon assay with A549 cells. This assay is based on transduction of cells with recombinant adeno-associated virus (AAV) expressing the Gaussia luciferase gene under control of the p40 promoter, which can be activated by helper proteins derived from adenoviruses and herpes viruses. Infection with the AAV vector is performed 24 h before infection with virus and treatment with peptides. Low basal expression of the reporter gene and strong induction dependent on the replication and spread of adenoviruses and herpes viruses allow determination of the potency of antiviral compounds (Koszinowski et al., 2016). As shown in Figure 3D, replication was dose-dependently inhibited by TAT-I24 (EC_50_ = 3.25 μM). The replicon assay was also performed with adenoviruses from two other subgroups. One member of the adenovirus subgroup D, adenovirus 19a/64 and adenovirus 4 belonging to subgroup E, was included. Interestingly, neither serotype responded to inhibition by the peptide at the concentrations used, indicating that the inhibitory activity of the peptide may depend on the mode of virus entry (Figure 3D). The TAT peptide had no effect on any of the adenoviruses tested (Supplementary Figure S3A). Cidofovir was included as a positive control for the replicon assay (Supplementary Figure S3B).

### TAT-I24 Inhibits Replication of Murine Cytomegalovirus

**Figure 4:**
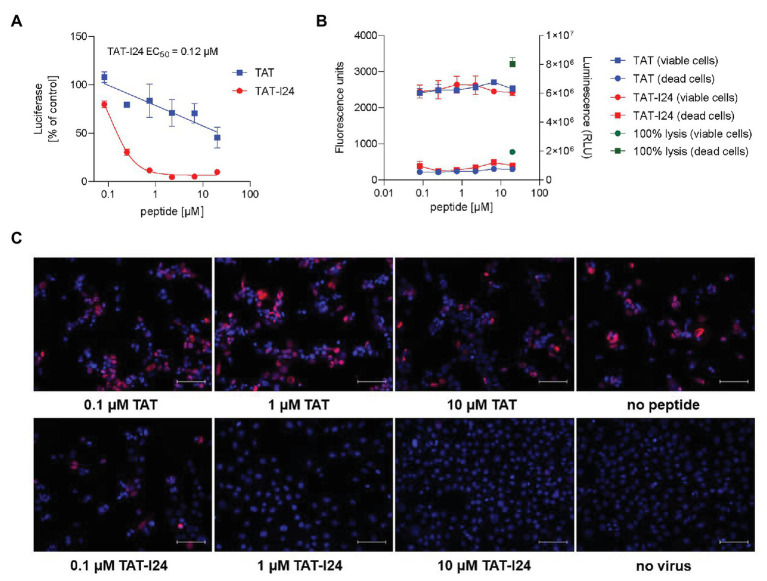
Inhibition of MCMV replication by TAT-I24. **(A)** NIH/3T3 cells were treated with increasing concentrations of TAT (48–60) or TAT-I24 and infected with murine CMV virus expressing firefly luciferase at a MOI of 0.5. After 72 h, luciferase was determined in the cell lysates. Results shown are mean ± SD from duplicate wells expressed as % of vehicle control. **(B)** Viable cells (fluorescence) and dead cells (luminescence; RLU = relative light units) in the presence of increasing concentrations of TAT or TAT-I24 determined by the cytotoxicity assay in triplicates. As positive control, 100% lysed cells are shown. **(C)** Microscopic examination (20 × objective) of NIH/3T3 cells infected with MCMV S-mCherry-SCP in the presence of increasing concentrations of TAT (48–60) or TAT-I24. Nuclei were stained with DAPI. Scale bars indicate 100 μm.

As an example of an enveloped double-stranded DNA virus, murine CMV (MCMV) was assessed for sensitivity to TAT-I24. NIH/3T3 cells were infected with MCMV expressing firefly luciferase (Trilling et al., 2011) in the presence of increasing concentrations of TAT or TAT-I24. Luciferase was determined from cell lysates 72 h after infection. The peptide TAT-I24 dose-dependently inhibited luciferase expression with EC_50_ values between 0.1 and 0.3 μM, while the TAT peptide only partially decreased cellular luciferase levels at higher concentrations (Figure 4A). As with the other types of virus, I24 without the TAT fusion peptide could not inhibit luciferase expression (Supplementary Figure S4A). Inhibition of MCMV replication by TAT-I24 was further confirmed using a plaque-formation assay (Supplementary Figure S4B). The potential cytotoxic effects of TAT and TAT-I24 on NIH/3T3 cells were examined by incubation of cells with increasing concentrations of peptide for 72 h and analysis of viable and dead cells. Lysed cells were included as positive control. No cytotoxic effects by the peptides were observed (Figure 4B).

Inhibition of virus replication was further visualized with a murine CMV strain expressing a capsid fused to mCherry (MCMV S-mCherry-SCP) described by Bosse et al. (2012). In the presence of various concentrations of TAT peptide, cells producing virus particles were seen 72 h after infection, while in the presence of TAT-I24 at concentrations ≥1 μM only basal fluorescence signal was observed (Figure 4C).

**Figure 5:**
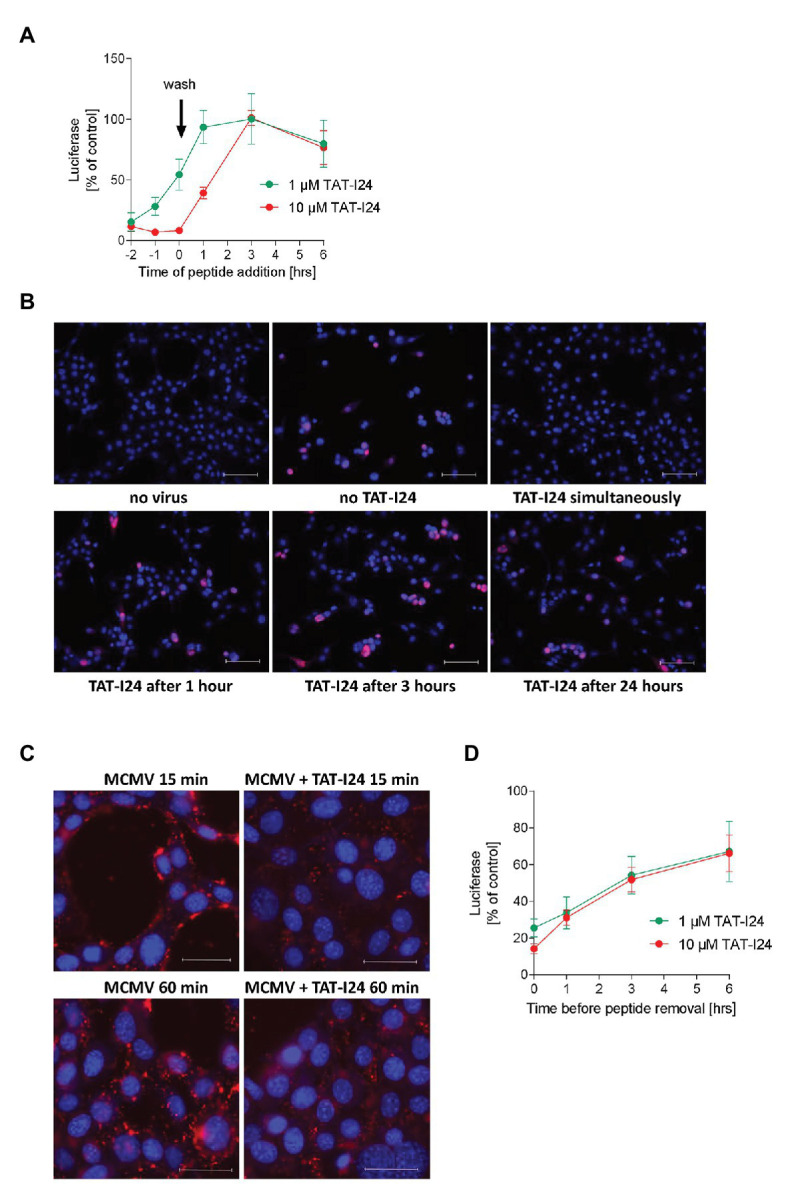
Inhibitory effect of TAT-I24 occurs at early steps upon virus entry. **(A)** NIH/3T3 cells were treated in duplicates with two different concentrations of TAT-I24 before or after virus adsorption at a MOI of 0.5 at +4°C at the indicated time points. Results shown are luciferase levels (mean ± SD) after 72 h from two independent experiments. **(B)** Microscopic examination (20 × objective) of NIH/3T3 cells infected with MCMV S-mCherry-SCP at +4°C in the presence of increasing concentrations of TAT-I24 applied at various time points after post-cold release. Nuclei were stained with DAPI. Scale bars indicate 100 μm. **(C)** NIH/3T3 cells were treated with 10 μM TAT-I24 or left untreated, followed by MCMV S-mCherry-SCP adsorption at +4°C with the aid of centrifugal enhancement. After 1.5 h, cells were washed with ice-cold medium and transferred to +37° before fixation with formaldehyde. Microscopic examination (40 × objective) of cells 15 and 60 min post-cold release. Nuclei were stained with DAPI. Scale bars indicate 40 μM. **(D)** NIH/3T3 cells were pretreated in duplicates with two different concentrations of TAT-I24 for the indicated time periods. Medium was then removed and cells were washed three times before virus infection and incubation for 72 h. Results shown are luciferase levels (mean ± SD) from two independent experiments.

To gain more insight into the mode of action, the effect of TAT-I24 at concentrations of 1 and 10 μM applied at different time points during MCMV infection was further studied in NIH/3T3 cells. To allow synchronization of the infection, cells were pre-treated with TAT-I24 and virus was adsorbed to cells by incubation at +4°C with the aid of centrifugal enhancement. After 1 h, cells were washed three times to remove unabsorbed virus and shifted to +37°C for 72 h. Preincubation or simultaneous incubation with TAT-I24 showed inhibition of luciferase levels, while a gradual loss of inhibition was observed when the peptide was added at later times after virus entry (Figure 5A). This observation is in accordance with reduced inhibition of luciferase gene expression by baculovirus in HEK293 cells and demonstrates that the peptide has to be present during virus infection. To confirm this time-dependence, MCMV S-mCherry-SCP was adsorbed to NIH/3T3 cells at +4°C with the aid of centrifugal enhancement and shifted to +37°C after three washes with pre-cooled medium. The peptide TAT-I24 at a concentration of 1 μM was added either during virus adsorption or at various time-points after temperature shift to +37°C. After 72 h, microscopic examination of cells producing virus particles was performed. TAT-I24 was already unable to inhibit MCMV replication when added 1 h after cold release, confirming that the peptide acts at early steps upon virus entry (Figure 5B).

Since the peptide was ineffective when added after infection with MCMV, virus entry was examined as a possible point of inhibition. MCMV S-mCherry-SCP was adsorbed to NIH/3T3 cells in the absence and presence of TAT-I24 at +4°C with the aid of centrifugal enhancement. After 1.5 h, cells were washed with ice-cold medium followed by a temperature shift to +37°C. Cells were fixed 15 and 60 min post-cold release. As shown in Figure 5C, overall fluorescence by mCherry-labeled viral capsids is clearly reduced in cells incubated with TAT-I24, indicating that the peptide indeed affects virus entry (Figure 5C).

To determine the consequence of removing the peptide from the cells, TAT-I24 was added at concentrations of 1 and 10 μM to NIH/3T3 cells for various time periods before removal of medium and three times washing of cells prior to virus adsorption. Interestingly, a gradual loss of the inhibitory effect was observed the longer the cells were preincubated with peptide before removal of medium and washing of the cells. This indicates that the inhibitory effect may be dependent on the simultaneous attachment of peptide and virus to the cell surface (Figure 5D).

**Figure 6:**
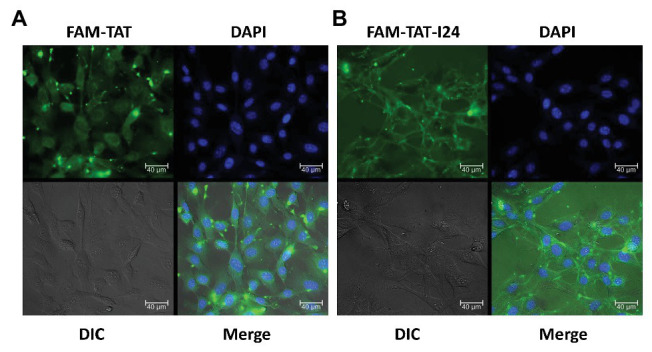
TAT-I24 is predominantly membrane-localized. NIH/3T3 cells were grown on poly-l-lysine coated chamber slides and allowed to attach overnight. Cells were then treated with 20 μM FAM-TAT **(A)** or FAM-TAT-I24 **(B)** for 1 h before fixation of cells with formaldehyde. The peptide FAM-TAT stains the cytosol and nucleoli, while FAM-TAT-I24 exhibits membrane localization and a cytosolic, punctate pattern (40 × magnification). Scale bars indicate 40 μm.

Cellular localization of peptides was therefore analyzed with NIH/3T3 cells using TAT and TAT-I24, both synthesized as fluorescein (FAM)-conjugates. As FAM-TAT-I24 formed precipitates when added to the medium directly, the peptide was first diluted with PBS before addition to the cell culture medium. This dilution reduced precipitate formation, which is likely to be caused by the hydrophobicity of the peptide I24 and the fluorescein dye. The remaining precipitate was then removed by centrifugation. NIH/3T3 cells were then treated with peptides (20 μM each) for 1 h before fixation and examination by microscopy. Cells treated with FAM-TAT exhibited fluorescence in the cytosol and nucleoli. In contrast, FAM-TAT-I24-treated cells exhibited a membrane localization together with a punctate pattern in the cytosol, but clearly excluding the nuclei, indicating that TAT-I24 is primarily membrane-associated (Figure 6).

### TAT-I24 Inhibits Replication of Human Cytomegalovirus

**Figure 7:**
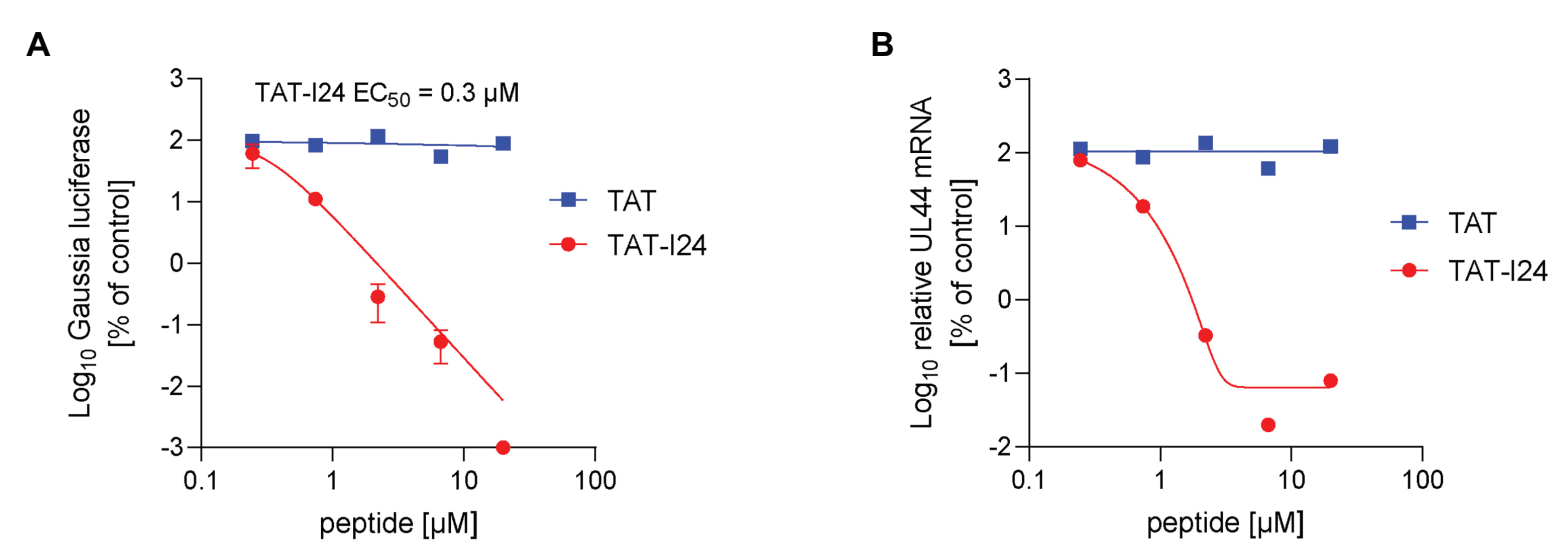
Inhibition of HCMV replication by TAT-I24. **(A)** MRC-5 cells were transduced with recombinant reporter construct AAV-GLuc-3B and treated on the next day with TAT (48–60) or TAT-I24 and infected with the HCMV strain TB40 at a MOI of 0.2. Results shown are Gaussia luciferase levels in the supernatants (mean ± SD from triplicate wells) 3 days after infection expressed as % of vehicle-treated cells. **(B)** Total RNA was isolated from cells from the replicon assay. Results shown are UL44 mRNA levels normalized to GAPDH mRNA levels expressed as % of control.

As MCMV was inhibited by TAT-I24, human cytomegalovirus (HCMV) was tested for its sensitivity to the peptides using a replicon assay performed with human MRC-5 fibroblast cells and recording of Gaussia luciferase 3 days after infection. The peptide TAT-I24 caused potent dose-dependent inhibition of HCMV replication over several log-steps with calculated EC_50_ values between 0.1 and 0.6 μM, while the TAT peptide alone had no effect (Figure 7A). Ganciclovir was included as a positive control in the same experiment (Supplementary Figure S5). HCMV transcript levels from lysates from the replicon assay were also strongly downregulated by TAT-I24, reflecting the results obtained with the replicon assay (Figure 7B). No effect on GAPDH mRNA levels was observed except at the highest concentration of 20 μM, where also some reduction of GAPDH mRNA levels by TAT-I24 was seen. However, at this concentration, UL44 mRNA levels were already at the lower detection limit of the real-time PCR.

### TAT-I24 Inhibits Replication of Herpes Simplex Viruses

**Figure 8:**
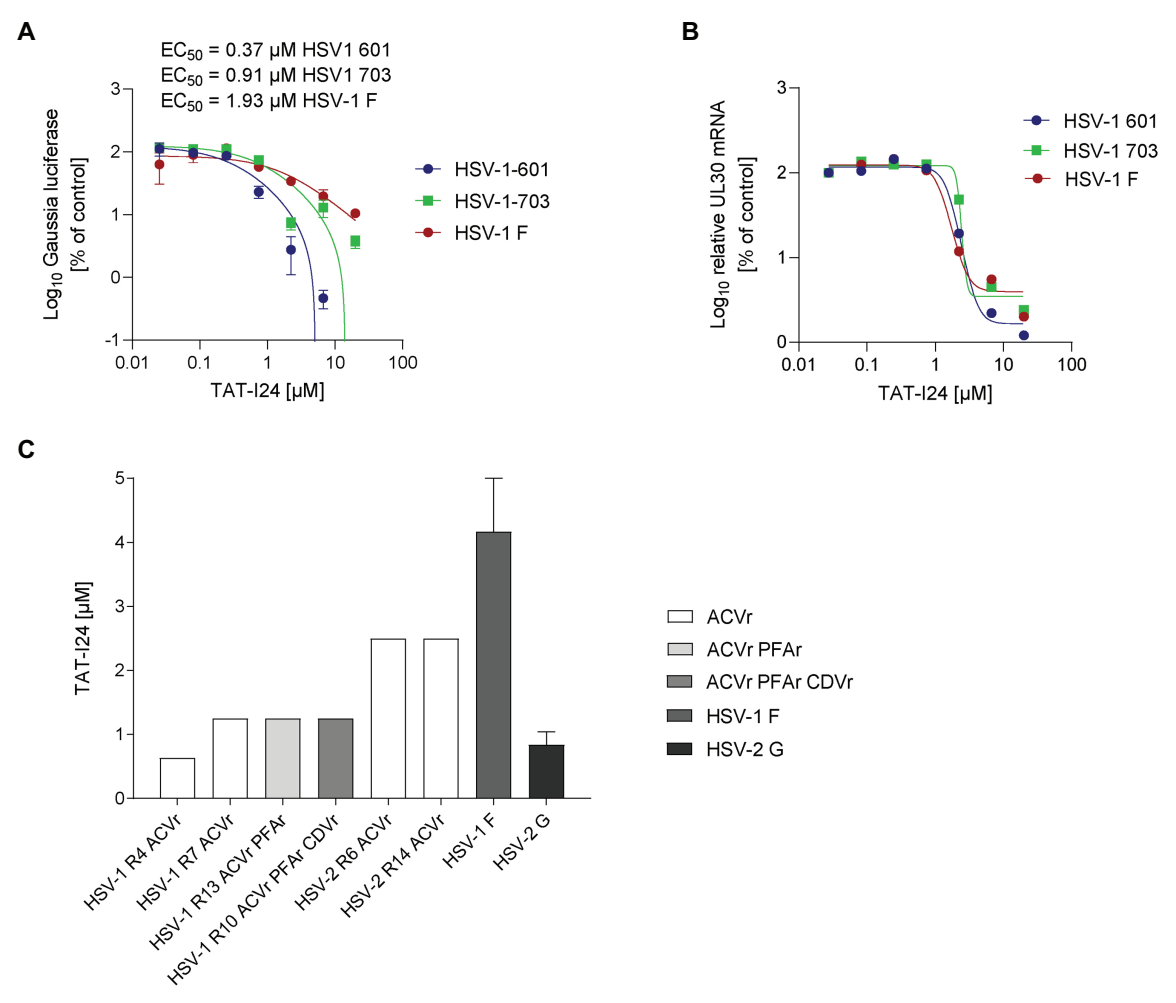
TAT-I24 potently neutralizes drug-resistant clinical isolates of HSV-1 and HSV-2. **(A)** Vero cells were transduced with recombinant reporter construct AAV-GLuc-3B for 24 h and infected on the next day with three different HSV-1 strains in the presence of increasing concentrations of peptides. Results shown are Gaussia luciferase levels in the supernatants (mean ± SD from triplicate wells) in % of vehicle-treated cells. **(B)** Total RNA was isolated from cells from the replicon assay. Results shown are UL30 transcript levels normalized to 18S rRNA expressed as % of vehicle-treated control. **(C)** The neutralizing capacity of TAT-I24 toward drug-resistant clinical isolates was assessed by endpoint dilution. Serial dilutions of TAT-I24 ranging from 0.08 to 10 μM were incubated with a viral load of 100 TCID_50_ of the indicated HSV-1 or HSV-2 isolates for 1 h at 37°C in cell culture medium. The TAT-I24/virus inoculum was applied to Vero cell monolayers grown in 96-well microtiter plates. The cytopathic effect (CPE) was scored after 48 h of incubation. The TAT-I24 concentration required for complete neutralization of the virus was defined as the neutralizing titer. ACVr/CDVr/PFAr: acyclovir−/cidofovir−/foscarnet-resistant.

Another member of the Herpesviridae, herpes simplex virus (HSV), was then assessed for inhibition by TAT-I24. A replicon assay was employed to determine sensitivity of HSV-1 isolates to TAT-I24. The strain HSV-1 F, and two isolates the aciclovir (ACV)-resistant HSV-1 703 and the aciclovir-sensitive isolate HSV-1 601, were tested. ACV was included as a control (Supplementary Figure S6A). Replication of all three HSV-1 strains was inhibited by TAT-I24 in a dose-dependent manner with EC_50_ values between 0.2 and 2 μM (Figure 8A). Again, the TAT peptide alone was ineffective in inhibiting reporter gene expression (Supplementary Figure S6B).

To exclude any effect of the peptide on the AAV vector itself, cells from the same wells were lysed for RNA isolation and expression of the UL30 gene was determined by real-time PCR and normalized to 18S ribosomal RNA (Kessler et al., 2000; Korom et al., 2008). As with CMV, inhibition of HSV-1 replication by TAT-I24 is also reflected at the level of viral mRNA, while the TAT peptide alone does not affect viral gene transcription (Figure 8B; Supplementary Figure S6C).

Finally, the neutralizing efficacy of TAT-I24 was assessed by an endpoint dilution assay with clinical HSV isolates, including ACV‐ and multi-drug-resistant clinical HSV-1 or HSV-2 isolates with additional resistance toward foscarnet (PFA) or cidofovir (CD) as described earlier (Krawczyk et al., 2013). Additionally, TAT-I24 was also tested on laboratory strains HSV-1 F and HSV-2 G, both susceptible to ACV. The peptide TAT-I24 completely neutralized ACV and multi-drug resistant clinical HSV-1 and HSV-2 isolates at a concentration ranging from 0.63 to 4.17 μM. The drug-resistant clinical isolates were neutralized at similar concentrations as laboratory strains susceptible to ACV. Neutralization efficacy of TAT-I24 was entirely independent of the clinical drug resistance status for all of the six clinical isolates (Figure 8C).

Replication of varicella zoster virus (VZV), another member of the Herpesviridae, was also inhibited by TAT-I24, albeit at higher concentrations compared to HSV and CMV (Supplementary Table S1; Supplementary Figure S8).

### Inhibition of Other Double-Stranded DNA Viruses by TAT-I24

**Figure 9:**
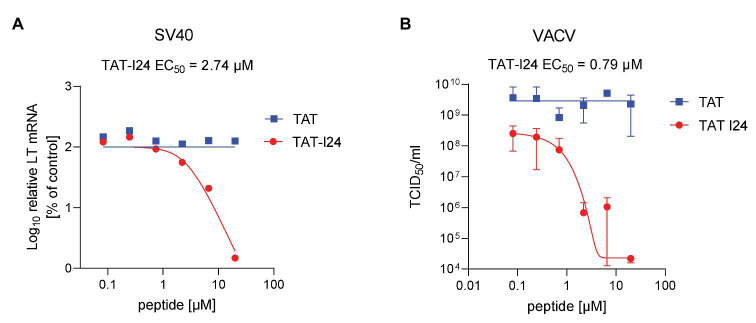
Inhibition of SV40 and vaccinia virus replication by TAT-I24. **(A)** Dose-dependent inhibition of large T (LT) antigen transcript levels in Vero cells infected with SV40 at a MOI of 2 and incubated with peptides TAT (48–60) or TAT-I24 for 3 days. Results shown are LT mRNA levels normalized to 18S rRNA determined by quantitative real-time PCR expressed as % of vehicle-treated control. **(B)** Dose-dependent inhibition of VACV wt replication by TAT-I24 determined by limited dilution of lysates from CV-1 cells infected with VACV wt in the presence of increasing concentrations of peptides. Data shown are mean ± SD from two independent experiments.

A member of the Polyomaviridae, the non-enveloped virus simian virus 40 (SV40), was tested for its sensitivity to the peptides. When infection was performed in the presence of TAT-I24, a dose-dependent decrease of large T antigen transcript levels was observed with EC_50_ values between 1 and 3 μM and approximately 98% inhibition compared to untreated cells at the highest concentration tested (20 μM). Again, the TAT peptide had no inhibitory effect on large T antigen mRNA levels (Figure 9A).

Sensitivity of vaccinia virus (VACV wt), an enveloped DNA virus exclusively replicating in the cytosol, was determined by infection of CV-1 cells in the presence of increasing concentrations of TAT or TAT-I24. While TAT-I24 caused potent dose-dependent inhibition of VACV wt replication over several log-steps, the TAT peptide alone had no effect (Figure 9B). No effect of DMSO or TAT-I24 on mock infected CV-1 cells was observed. Although TAT-I24 at higher dosages (20 and 6.6 μM) had some effects on CV-1 cell attachment, it clearly blocked vaccinia virus replication as determined from titration of both cell lysates and supernatants.

### TAT-I24 Is Less Effective Against RNA Viruses

**Figure 10:**
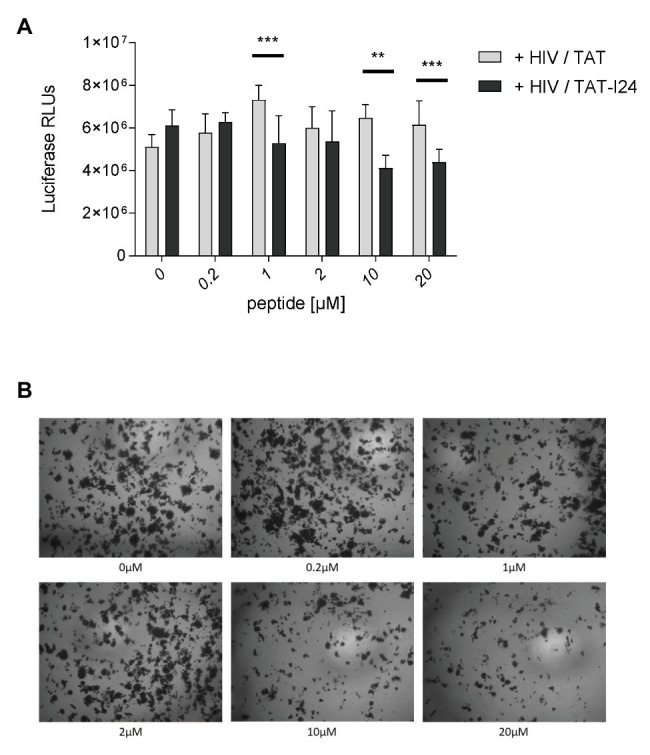
Impact of TAT-I24 on HIV replication. HIV strain NL4-3 was pre-incubated with the indicated concentrations of TAT or TAT-I24 prior to infection of Jurkat cells (0.05 MOI). Six days post-infection supernatants were tested for the presence of infectious HIV particles using TZM-bl reporter cells. **(A)** Luciferase activity 48 hours post-infection of the reporter cells. **(B)** X-Gal staining of TZM-bl reporter cells infected with TAT-I24 pre-treated viruses. Multiple T test was used for statistical analysis (^**^p ≤ 0.01 and ^***^p ≤ 0.001). RLU, relative light units.

To analyze the effect of peptides on replication of the retrovirus human immunodeficiency virus-1 (HIV-1), Jurkat cells were infected with the laboratory strain NL4-3 at a MOI of 0.05 and pre-incubated with increasing concentrations of TAT or TAT-I24. Six days after infection, supernatants were transferred to TZM-bl reporter cells harboring the luciferase gene under control of the HIV-1 LTR promoter. Forty-eight hours later, luminescence was measured to quantify infectious viral particles. A moderate but significant reduction of the infection efficiency was observed with supernatants from cells, which were infected with TAT-I24 pre-incubated virus (Figure 10A). This was also visible in X-Gal stained HIV infected cells (Figure 10B). In the absence of virus, no inhibitory effect of TAT-I24 on reporter gene expression was observed (Supplementary Figure S7).

Of the RNA viruses tested for their sensitivity to TAT-I24, influenza A virus was not inhibited at the concentrations used, while only a partial reduction of respiratory syncytial virus RNA by TAT-I24 was observed (Supplementary Table S1; Supplementary Figure S9).

## Discussion

The peptide TAT-I24 inhibits growth of several double-stranded DNA viruses in mammalian cells, including herpes simplex virus, cytomegalovirus, adenovirus type 5, vaccinia virus, and SV40 polyomavirus. RNA viruses and the retrovirus HIV-1 are less sensitive to inhibition by the peptide. Our data indicate that TAT-I24 inhibits an early step of the viral replication cycle at the level of viral entry and viral gene expression. Baculovirus in the presented experimental setting did not replicate in mammalian cells but transduction was still clearly inhibited by TAT-I24, indicating that the peptide acts at a step before the onset of virus replication. The propensity for inhibition of DNA viruses as opposed to others indicates that the type of viral genome accounts for the sensitivity to the peptide. However, enveloped DNA viruses responded better than non-enveloped viruses, such as adenovirus type 5 and SV40 virus, which required higher concentrations of TAT-I24 for inhibition. Any evidence of direct binding of the peptide to the viral envelope and/or of an effect on membrane structures will be of importance in elucidation of the mechanism of action. While active against DNA viruses, replication of RSV and HIV was only partially inhibited by TAT-I24.

Although the precise mode-of-action is not yet fully resolved, the peptide most likely acts at early stages of infection upon or immediately after virus entry. This is supported by the observation that TAT-I24 shows the highest levels of inhibition when added during virus infection but becomes less active when applied few hours after infection. In addition, TAT-I24 clearly reduced viral entry into cells infected with MCMV.

However, the preference for inhibiting double-stranded DNA viruses may not be explained solely by a block of viral entry. A direct, inhibitory effect on gene expression by I24 and TAT-I24 on transfected DNA supports the hypothesis that the peptide could directly affect viral gene expression. A peptide with a similar cysteine-containing motif, 6N40, was identified by phage screening against the papillomavirus 11 E2 protein. This peptide inhibited transcription and replication of papillomavirus through interfering with the DNA binding activity of the E2 protein. However, no direct binding of 6N40 to DNA could be demonstrated (Deng et al., 2004). In their study, a second peptide (2N18) with a sequence even more similar to I24 is listed, but no further data provided. It is therefore possible that these peptides act in a similar way to I24. Further modification of amino acids of I24 coupled to the TAT peptide should help to identify residues, which are essential for the inhibitory effect.

TAT-I24 is predominantly membrane-localized with no nuclear staining visible under these conditions. However, it cannot be excluded that low amounts of peptide move to the nucleus but are not detected by microscopy. Localization studies will therefore be extended to other fusion partners of I24 such as the SV40 NSL peptide, which would be expected to result in nuclear localization of the peptide (Kalderon et al., 1984). However, due to its hydrophobicity, I24 may trap the fusion peptide in the cellular membrane and could thus prevent its movement to the nucleus.

The TAT-I24 peptide had effects in viral infection models, which were remarkably similar to those of I24 alone in the cellular transfection model, indicating that features of I24 and some of the features of the TAT peptide lead to enhanced activity. The TAT peptide is derived from the basic region of the HIV-1 transactivator protein (Green and Loewenstein, 1988). It has been shown to enter cells by diffusion and endocytosis and is a well-known cell penetrating peptide (reviewed by Rizzuti et al., 2015). However, the TAT peptide not only mediates cell penetration but also binds to different molecules. For instance, the TAT peptide has been shown to electrostatically interact with DNA and can enable transfer of plasmid DNA into cells (Ignatovich et al., 2003; Ziegler and Seelig, 2007). The TAT peptide may therefore promote DNA-binding of I24 as the fusion TAT-I24 showed enhanced potency in inhibiting gene expression from the transfected plasmid DNA compared to I24 alone. Additionally, the TAT peptide can increase solubility of fusion partners and may therefore also improve the solubility of the hydrophobic I24 peptide, thereby enhancing its activity.

Another important feature of the TAT peptide is binding to heparan sulfate (HS), which acts as a cell-surface receptor of diverse cargoes, including viruses (Ziegler and Seelig, 2004; Richard et al., 2005; Christianson and Belting, 2014). The TAT peptide could therefore guide I24 to HS, which mediates attachment and penetration of several viruses, including herpes simplex virus, cytomegalovirus, or varicella-zoster virus, and also poxviruses and papillomaviruses (Zhu et al., 2011). Baculovirus has also been reported to bind *via* HS to mammalian cells (Duisit et al., 1999). In contrast, adenoviruses use different receptors, such as Coxsackie and Adenovirus receptors (CAR), CD46, or α(2–3)-linked sialic acid (Gaggar et al., 2003; Zhang and Bergelson, 2005; Nemerow et al., 2009). However, adenoviruses 2 and 5 have also been reported to attach to HS, indicating that HS can be used in addition to CAR for virus entry (Dechecchi et al., 2000). It is therefore possible that the effect of TAT-I24 on adenovirus 5 is associated with its binding to HS. Interestingly, adenovirus 4, which binds to CAR and adenovirus 19a/64, binding *via* sialic acid, did not respond to treatment with TAT-I24 at the concentrations used, providing support to the idea that HS contributes to the inhibitory effect of the peptide. The testing of additional types of adenoviruses and engineered cell lines allowing restricted receptor usage of adenoviruses (Zhang et al., 2017) is therefore the subject of ongoing studies. These comparative adenovirus-based approaches may provide further insights into the mechanistic basis of TAT-I24 inhibition of DNA virus replication.

Inhibition of herpes simplex virus entry by the TAT peptide has been described (Bultmann and Brandt, 2002; Akkarawongsa et al., 2008), and further modification of the TAT peptide by addition of a C-terminal cysteine residue improved this inhibitory activity. Upon pre-treatment with the peptide, cells became resistant to virus infection. Moreover, TAT-C has been shown to inactivate virions in solution (Bultmann et al., 2007). The inhibitory effect of TAT-C was also observed *in vivo* using a herpes simplex-induced keratitis model (Jose et al., 2013). However, the TAT peptide alone in the present study showed no or only very limited effects on any of the virus systems tested. We also tested a TAT (47–57) variant with a C-terminal cysteine residue and found only partial inhibition at the highest concentration of 20 μM in the Baculovirus-Luc expression system. The ability of the fusion of I24 and the TAT peptide to neutralize various types of viruses at micromolar to submicromolar concentrations indicates that features of both TAT and I24 contribute to the inhibitory effect in different viral infection models.

Other cell penetrating peptides have been fused to I24 with inhibitory effects at higher concentrations compared to the TAT peptide (*unpublished observations*). Another fusion partner, SV40 NLS, which is derived from the SV40 large T antigen, was chosen as it contains a basic motif, which resembles a well-known nuclear localization signal and has been used to promote DNA transfer to the nucleus (Kalderon et al., 1984; Zanta et al., 1999). However, fusion of SV40 NLS to I24 caused dose-dependent inhibition of reporter gene expression by baculovirus. Potency was similar to TAT-I24, although the overall extent of inhibition was lower. These data indicate that I24 fusion partners other than the TAT peptide can also exert an inhibitory effect.

**Figure 11:**
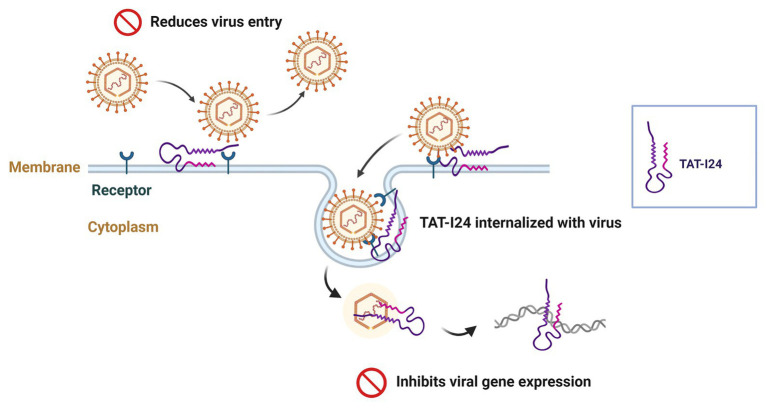
Proposed model of a dual mode-of-action of TAT-I24: while the peptide reduces overall viral entry, the remaining bound virus can also be internalized together with the peptide, which subsequently binds to viral DNA upon uncoating and reduces viral gene expression. Created with BioRender.com.

A proposed model of the mode-of action of TAT-I24 is depicted in Figure 11 and indicates intervention at two points. While overall viral entry is reduced by the peptide, TAT-I24 can be internalized with the remaining bound virus, where it interacts with the viral DNA upon uncoating, leading to inhibition of viral gene expression (Figure 11). However, further analyses are necessary to fully elucidate this inhibitory mechanism.

The peptide therefore represents a promising approach to develop a novel drug candidate as broad-spectrum antiviral agent against double-stranded DNA viruses, in particular herpes simplex virus and cytomegalovirus.

## Data Availability Statement

The raw data supporting the conclusions of this article will be made available by the authors, without undue reservation.

## Author Contributions

ZR, AR, and HHe planned and performed the ADV, HSV, and CMV replicon assays. KH planned and performed the vaccinia virus experiments. AK and LS planned and performed the HSV-1 and HSV-2 neutralization assays. MW and HS planned and performed the HIV-1 experiment. VK-F planned and performed the influenza, VZV, and RSV experiment. MD assisted with microscopy. WE and RG provided the Baculovirus system. HHa performed the transfection, baculovirus, ADV, and MCMV experiments. HHa, ZR, AK, MW, KH, RG, and HHe wrote the manuscript. All authors read and approved the final manuscript.

### Conflict of Interest

HHa is the inventor of patent WO2019/057973 and holds 100% of the shares of Pivaris BioScience GmbH. ZR is co-inventor of patent WO2017/129822.

The remaining authors declare that the research was conducted in the absence of any commercial or financial relationships that could be construed as a potential conflict of interest.
