# Supplementary material for: A Novel, Broad-Acting Peptide Inhibitor of Double-Stranded DNA Virus Gene Expression and Replication
Source: Front Microbiol. 2020 Nov 17;11:601555. doi: 10.3389/fmicb.2020.601555 (PMC7705112; doi:10.3389/fmicb.2020.601555)
Supplement: Supplementary file 1 [file Data_Sheet_1.docx]

***Supplementary Information***

***Figure S1***

**Supplementary Figure S1**

**Inhibition of reporter gene expression is less pronounced when transfection is performed using lipofection**

HEK293 cells were seeded at a density of 2.5 x 10^4^ cells/well in 96-well plates. On the next day, 100 ng of plasmid encoding firefly luciferase under control of the CMV promoter were added to 15 µl serum- and antibiotic-free medium followed by addition of 0.6 µl I24 peptide (10 mM and 1 mM). Then, 15 µl medium containing 3.75 µl lipofectamine were added to the DNA-peptide mixture. After an incubation for 15 min at room temperature, 270 µl of medium were added and 140 µl added to each well of duplicate wells. After 5 hrs incubation at 37°C, 100 µl medium containing 10% foetal calf serum were added. Twenty-four hours later, cells were lysed and luciferase determined using Luciferase Assay Reagent. Transfection with Superfect is described in the Material & Methods section of the main article. Results shown are luciferase levels (mean ± SD from duplicate wells) as % of vehicle-control.

***Figure S2***

**Supplementary Figure S2**

**TAT-I24, but not I24, inhibits gene expression of adenovirus type 5**

HEK293 cells were seeded at a density of 2 x 10^4^ cells/well of a 96-well plate and infected on the next day with adenovirus particles expressing luciferase/GFP (MOI = 0.2) in the presence of increasing concentrations of I24 or TAT-I24. Luciferase from cell lysates was recorded 24 hours after infection. Results shown are luciferase levels expressed as % of control from duplicate wells. Luciferase was detected using Luciferase Assay System as described in Materials & Methods of the main article.

***Figure S3***

***A***

***B***

**Supplementary Figure S3**

**Effect of TAT and cidofovir on replication of adenovirus 5, adenovirus 4 and adenovirus 19a/64**

A549 cells were transduced with recombinant reporter construct AAV-GLuc-3B and treated on the next day with increasing concentrations of TAT peptide (A) or cidofovir (B) and infected with adenovirus 4, 5 or 19a/64. Gaussia luciferase levels in the supernatants expressed as % of vehicle-treated control are shown (mean ± SD from triplicate wells). Experiments were performed as described in the Materials & Methods section of the main article.

***Figure S4***

**A**

**B**

**Supplementary Figure S4**

**TAT-I24, but not I24, inhibits gene expression of MCMV**

A, NIH/3T3 cells were seeded at a density of 2 x 10^4^ cells/well of a 96-well plate and treated on the next day with increasing concentrations of I24 or TAT-I24 and infected with MCMV (MOI = 0.5). After 72 hrs, luciferase was determined from cell lysates. Results shown are luciferase levels expressed as % of control from duplicate wells. Luciferase was detected using Luciferase Assay System as described in Materials & Methods of the main article. B, Murine embryonic fibroblasts were pre-treated for one hour with increasing concentrations of TAT-I24 and infected with MCMV (BAC derived wt Smith strain; (Jordan et al., 2011)). Plaque formation was determined after 5 days. Number of plaques in % of untreated controls are shown.

**Reference:**

Jordan, S., Krause, J., Prager, A., Mitrovic, M., Jonjic, S., Koszinowski, U. H., et al. (2011). Virus Progeny of Murine Cytomegalovirus Bacterial Artificial Chromosome pSM3fr Show Reduced Growth in Salivary Glands due to a Fixed Mutation of MCK-2. *J. Virol.* 85, 10346–10353. doi:10.1128/JVI.00545-11.

***Figure S5***

**Supplementary Figure S5**

**Effect of ganciclovir on replication of human CMV**

MRC-5 cells were transduced with recombinant reporter construct AAV-GLuc-3B and treated on the next day with ganciclovir and infected with the HCMV strain TB40 at a MOI of 0.2. Results shown are Gaussia luciferase levels in the supernatants (mean ± SD from triplicate wells) 3 days after infection expressed as % of vehicle-treated cells.

***Figure S6***

**A**

**B**

**C**

**Supplementary Figure S6**

**Effect of aciclovir and TAT on replication of herpes simplex viruses**

Vero cells were transduced with recombinant reporter construct AAV-GLuc-3B and treated on the next day with increasing concentrations of aciclovir (A) or TAT peptide (B). Cells were then infected with three different HSV-1 isolates, the laboratory strain HSV-1 F, the aciclovir-sensitive isolate HSV-1 601 and the aciclovir-resistant isolate HSV-1 703. Results shown are Gaussia luciferase levels in the supernatants (mean ± SD from triplicate wells) in % of vehicle-treated cells. The resistance of the HSV-1 isolate 703 to aciclovir was confirmed by this assay. No inhibition by the TAT peptide on reporter gene expression was observed. C, RNA was isolated from cells from the replicon assay treated with TAT peptide and HSV-1 transcripts analysed by real-time PCR. Results shown are UL30 transcript levels relative to the 18S rRNA expressed as % of vehicle-treated control. No effect of the TAT peptide on UL30 mRNA levels was observed, confirming the results from the replicon assay. Experiments were performed as described in the Materials & Methods section of the main article.

***Figure S7***

**Supplementary Figure S7**

**TAT-I24 does not inhibit reporter gene expression in non-infected TZM-bl reporter cells**

Luciferase activity of TZM-bl reporter cells assayed 48 hours after treatment with TAT and TAT-I24 in the absence of infectious HIV-1 particles. No inhibition of the reporter gene by TAT or TAT-I24 was observed. Experiments were performed as described in the Materials & Methods section of the main article.

***Table S1* Assessment of sensitivity of other viruses to TAT-I24**

| **Virus type** | **Genome** | **TAT-I24** | **TAT** |
| --- | --- | --- | --- |
| Varicella zoster virus | dsDNA | CPE: 6.6 µM (ACV: 4 µM) | no inhibition |
| Influenza A Virus | ssRNA | Plaque assay: no inhibition | no inhibition |
| Respiratory syncytial virus A | ssRNA | CPE: > 20 µM | no inhibition |

**Description of the experiments:**

**Varicella zoster virus (VZV):** Human fibroblasts were pre-treated with TAT (48-60) or TAT-I24 and infected with varicella zoster virus (VZV) for one hour. Aciclovir (ACV) was included as control. After removal of unabsorbed virus by washing, medium containing peptide dilutions was added to cells. After three days, the cytopathic effect was analysed by microscopy.

**Influenza A** **virus**: The effect of peptides on influenza A replication was determined by plaque-formation assay. MDCK cells were infected with influenza A PR8 in the presence of peptides or oseltamivir as positive control for three days. The peptides at the concentrations used did not affect replication of influenza A virus.

**Respiratory syncytial virus (RSV):** Human Hep2 cells were pre-treated with peptides and infected with cell-free RSV Stock (strain A2) for 2 hrs at 37°C. Medium was then removed and unabsorbed virus removed by washing. Medium containing peptide dilutions was then added to cells. After 3 days, the cytopathic effect was analysed by microscopy.

***Supplementary Figure S8***

**Supplementary Figure S8**

**Effect of TAT or TAT-I24 on VZV transcript levels**

VZV transcripts were analysed from lysates of infected cells by real-time PCR using ViroReal® VZV (RTGM20V; Ingenetix GmbH, Austria) and normalized to GAPDH mRNA. TAT-I24 reduced levels of VZV transcripts with a calculated EC_50_ of 3.3 µM.

***Supplementary Figure S9***

**Supplementary Figure S9**

**Effect of TAT and TAT-I24 on RSV infection**

Lysates from infected cells were examined for the presence of RSV RNA by real-time PCR using ViroReal® RSV (RTGM20V; Ingenetix GmbH, Austria) detecting the nucleocapsid protein gene of RSV and normalized to GAPDH mRNA. Calculated EC_50_ was 0.8 µM for TAT-I24 with 80% inhibition at the highest concentration (20 µM). Ribavirin was included as control.
